# Oxidative Stress Mediates Physiological Costs of Begging in Magpie (*Pica pica*) Nestlings

**DOI:** 10.1371/journal.pone.0040367

**Published:** 2012-07-10

**Authors:** Gregorio Moreno-Rueda, Tomás Redondo, Cristina E. Trenzado, Ana Sanz, Jesús M. Zúñiga

**Affiliations:** 1 Departamento de Zoología, Universidad de Granada, Granada, Spain; 2 Estación Biológica de Doñana, Consejo Superior de Investigaciones Científicas, Sevilla, Spain; 3 Servicio de Producción y Experimentación Animal, Centro de Instrumentación Científica, Universidad de Granada, Granada, Spain; Dalhousie University, Canada

## Abstract

**Background:**

Theoretical models predict that a cost is necessary to guarantee honesty in begging displays given by offspring to solicit food from their parents. There is evidence for begging costs in the form of a reduced growth rate and immunocompetence. Moreover, begging implies vigorous physical activity and attentiveness, which should increase metabolism and thus the releasing of pro-oxidant substances. Consequently, we predict that soliciting offspring incur a cost in terms of oxidative stress, and growth rate and immune response (processes that generate pro-oxidants substances) are reduced in order to maintain oxidative balance.

**Methodology/Principal Findings:**

We test whether magpie (*Pica pica*) nestlings incur a cost in terms of oxidative stress when experimentally forced to beg intensively, and whether oxidative balance is maintained by reducing growth rate and immune response. Our results show that begging provokes oxidative stress, and that nestlings begging for longer bouts reduce growth and immune response, thereby maintaining their oxidative status.

**Conclusions/Significance:**

These findings help explaining the physiological link between begging and its associated growth and immunocompetence costs, which seems to be mediated by oxidative stress. Our study is a unique example of the complex relationships between the intensity of a communicative display (begging), oxidative stress, and life-history traits directly linked to viability.

## Introduction

Evolutionary theory hypothesizes that many animal signals have to be costly in order to reliably indicate signaler quality [1]–[3]. The conspicuous begging displays given by offspring to solicit food from their parents are considered an expression of a genetic conflict of interests over the allocation of parental resources among dependent young. Parent-offspring conflict theory holds that optimal investment levels should differ for parents and offspring, with offspring attempting to obtain more resources than parents are selected to supply [4]–[6]. Showy begging displays may have evolved either as selfish attempts to influence parental decisions in scramble sibling competition for limited resources [7], [8] and/or as honest signals of need allowing parents to allocate food in relation to begging intensity [9]. In both cases, costly begging may limit offspring behavior, preventing runaway escalation of begging intensity over evolutionary time, and thus allowing a stable equilibrium for conflict resolution [9], [10]. Both evolutionary scenarios arrive at virtually the same predictions, namely that begging intensity should reliably covary with nutritional condition and entail a proportional cost [11]. There is good evidence that begging reliably signals short-term nutritional need [3]. And, with few exceptions [12], most theoretical models which aimed at explaining the evolution of honest, information-rich begging signals, conclude that a cost function that increases with increasing begging intensity and penalizes misrepresentation is essential for stability [13], [14], although other mechanisms (e.g. kin selection) may also contribute to keep signals honest at a relatively lower direct cost [15]–[17].

Nestlings may incur different types of direct costs when begging intensively. First, loud begging calls may attract eavesdropping predators to the nest and nestling jostling may increase brood conspicuousness [18]. While this cost may have limited absolute begging intensity over evolutionary time according to predation risk [19], nest predators typically kill all nestlings in a brood (not only cheaters) and parents may actively reduce nest vulnerability by silencing nestlings or defending them [20], irrespective of begging intensity. Therefore, it is unclear whether predation costs could stabilize honest, informative begging in multichick broods, as long as this cost must be higher for the chick that begs more than for its siblings [13], [21]. Second, nestlings may incur individual, physiological costs directly proportional to the duration and intensity of begging signals. Recent theoretical refinements emphasize that these marginal costs need not to be high at the honest equilibrium, but should be potentially high enough for cheaters giving exaggerated signals [15], [22]. Consequently, marginal costs can be measured only by experimentally manipulating nestlings into giving out-of-equilibrium signals, a long-overlooked fact that may have hampered early attempts to quantify begging costs empirically [23].

Begging usually involves vigorous posturing and calling [24] and considerable attentiveness [25], which implies elevated metabolic rates. Metabolism generates pro-oxidant substances (reactive oxygen/nitrogen species, RONS) [26]. RONS react with lipids, proteins and nucleic acids, with long-term negative consequences for cellular performance [27]. To prevent cellular damage caused by RONS, organisms display a number of anti-oxidant mechanisms, which include a set of enzymes [28]. When antioxidants cannot combat RONS, oxidative stress occurs, and cells may be damaged [27], [28]. Therefore, if begging favors the production of RONS above the antioxidant capacity of the organism, intensive begging will cause oxidative stress and damages in diverse tissues (e.g., brain, germ line, [29], [30]), which may result in a reduction of fitness.

Nestlings begging intensively may reduce their growth rate ([21], [23], [31]; but see [21], [32]) and immunocompetence [31], [33]. Both growth and immune response increase the release of RONS [34]–[37]. Hence, we hypothesized that intensive begging increases production of RONS, and that nestlings begging intensely reduce growth and immune response to avoid oxidative damage. Indirect evidence supports the idea that risk of oxidative stress limits the escalation of begging, as nestlings supplied with antioxidants (carotenoids and vitamin E) beg at elevated levels ([38], [39], but see [40]). However, no study so far has tested if nestlings begging at high rates incur elevated oxidative stress. Our goal was to test whether exaggerated begging increases the risk of oxidative stress and whether nestlings reduce growth and immunocompetence to maintain oxidative status. We experimentally forced magpie (*Pica pica*) nestlings into begging for long bouts during three consecutive days at the laboratory (high begging, HB nestlings), while their control nestmates were kept begging at a much shorter rate (low begging, LB nestlings) for the same amount of food. Magpie nestlings show reduced growth and immunocompetence when begging at high rates ([21], [41], unpubl. ms). Then, we estimated lipid peroxidation (levels of malondialdehyde, MDA) as an indicator of oxidative stress, as well as the antioxidant status of the chicks by determining the activity of the enzymes superoxide dismutase (SOD), glutathione reductase (GR), and glutathione peroxidase (GPX). We predicted that, other things being equal, intensive begging should increase the levels of MDA, but nestlings should attempt to maintain their oxidative balance by either increasing their antioxidant status and/or reducing other pro-oxidant processes such as growth or immune response.

## Materials and Methods

### General Methods and Experimental Design

The study was carried out during the spring of 2010, with a population of European magpies located at Santa Fe and Chimeneas (SE Spain). The study area is formed by a mix of farmlands, mainly cereals, with scattered olive and almond trees, where the magpies nest. Nests were inspected regularly to determine the exact date of hatching (day 0). We located 42 active nests with complete clutches, but high rates of nest destruction by local people, natural predation, and parasitism by great spotted cuckoos (*Clamator glandarius*) reduced sample size to 12 available unparasitized broods of an appropriate age. We used 32 nestlings (2 or 4 nestlings per nest, depending on brood size) when they were 9 d old, i.e. two days before the age at which they grow at the highest rate [42]. This ensured that nestlings were growing at maximum rates on the second day of the laboratory experiment. In the evening of the day before the start of the experiment, we took the nestlings, leaving at least two chicks in the nest to prevent parental desertion. Chicks were placed in a warm chamber and taken to a laboratory at the Animal Experimentation Unit in the University of Granada, transportation lasting about 30 min. On that evening, nestlings were fed *ad libitum*. Nestlings were maintained in the laboratory for three days, and when the experiment ended, nestlings were fed *ad libitum* again and returned back to their nests during the morning. No nestling died or suffered damage during the study, and parents accepted all nestlings returned back to their nests.

We randomly assigned one member of each pair of nestmates of a similar body mass to either a high begging (HB, *n* = 16) or a low begging (LB, *n* = 16) treatment. Four nests contributed two pairs to the experiment. During the three days of the experiment, each nestling was maintained isolated in a cloth cup simulating a nest, at a constant temperature of ca. 36°C. While resting, nestlings were covered by a duster, simulating brooding by the mother, which precluded nestlings from begging between trials. During each feeding, for the three days of the experiment, nestlings were stimulated to beg by using a human word (“toma”) at which nestlings were previously trained to respond the day before the experiment began. Low begging (LB) nestlings were fed immediately after gaping, while HB nestlings were stimulated to beg for 1 min before being fed. Therefore, experimental manipulation caused HB nestlings to beg for considerably longer bouts than their LB nestmates. Considering that magpie nestlings beg for 7–18 s/hour on average [21], [43], most nestlings in a brood tend to beg in response to a feeding visit by adults [44], and mean rates of adult visits at the age of nestlings used here (10–12 days) are 5.5 visits/hour [41], a gross estimate of natural begging rates is ca. 40–100 s/hour. Therefore, hourly begging rates in natural nests may not be strikingly different from those in the HB group. Begging behavior was recorded with a digital camera Handycam HDR-XR155E (Sony). From video recordings, we measured the time each nestling spent begging by continuous sampling, using the JWatcher 1.0 software [45]. To estimate total time begging for each HB and LB nestling, we randomly selected five trials on days 1 and 3 of the experiment. Time begging measured from different sets of five trials were significantly correlated (*r* = 0.78; *P*<0.001), suggesting that estimates were repeatable. Technical difficulties with recording three LB nestlings reduced sample size for the variable “time begging” to 13 LB nestlings.

Each morning, nestlings were weighed before being fed with a digital balance (accuracy 0.01 g) and the mass of the first fecal sac discounted, to calculate body mass. We estimated the food to be ingested by nestlings during each experimental day according to their mass, following the allometric relationship between daily food consumed and daily growth [46]: daily food to be consumed  = 0.98×M^0.814^, where M is nestling body mass in grams. Daily food intake was divided in 14 equal portions corresponding to the 14 feeding trials; any deviations from expected food intake during an hour were compensated for in subsequent trials. Food was composed of moistened puppy chow with a high protein content (ca. 50% of dry weight) and enriched with vitamins A, D3, and E, calcium and phosphorus. Feces excreted by nestlings were weighed. Mass gained was estimated as the difference in body mass between the first and the last day of the experiment.

We also measured how the experimental treatment affected the ability to mount a general inflammatory response. The third day of the experiment, at 20∶00 h in the evening, we injected into the left patagium of each chick 0.4 mg of phytohaemagglutinin (PHA-P, L-8754, Sigma Aldrich) diluted in 0.08 ml of isotonic phosphate buffer [47]. PHA-P is an innocuous protein that provokes a general inflammatory response mediated by T-cells [48], although other components of the immune system are also involved [49], [50]. Previously, we had measured (three times) the patagium thickness with a pressure-sensitive micrometer (Mitutoyo; accuracy: 0.01 mm). On the next day, at 8∶00 h (12 h later), we again measured the patagium thickness, calculating the inflammatory response as the difference between the second and first measurements. Six hours is enough to detect a response to PHA [49], [51]. The highest the swelling of the patagium in response to the mitogen, the highest the T-cell mediated immune capacity of the nestling is.

### Biochemical Analyses

When the experiment ended, we extracted approximately 200 µl of blood from the brachial vein during the last morning the chicks were in the lab. By taking blood samples after nocturnal fast, problems with effects of digestion on biochemical traits are avoided [52]. Blood samples were centrifuged at 3000 *g* during 10 min, and plasma was separated from red cells. Red blood cells were cleaned with 9 g/L NaCl solution and centrifuged again, removing the supernatant. Then, samples of erythrocytes were diluted in distilled water (proportion of 1∶4), provoking cell lysis, and the hemolysate was frozen at −80°C until the analyses. All enzymatic assays were carried out at 25±0.5°C using a PowerWavex microplate scanning spectrophotometer (Bio-Tek Instruments, USA) in duplicate in 96-well microplates (UVStar®, Greiner Bio-One, Germany). We measured the activity of Superoxide dismutase (SOD), Glutathione peroxidase (GPX), and Glutathione reductase (GR) following standardized techniques (Method S1). Enzymatic activity of SOD is expressed in U/ml of hemolysate, while for GPX and GR it is expressed as mU/ml of hemolysate. The soluble protein content of the solutions was determined by the Bradford method [53], using bovine serum albumin as the standard. Lipid-peroxidation levels were determined by quantifying the concentration of thiobarbituric-acid-reacting substances (TBARS), expressed as nmol malondialdehyde (MDA) per ml of hemolysate [54]. Malondialdehyde results of the reaction between RONS and polyunsaturated lipids, and it is frequently used as a marker of oxidative stress. For all the biochemical variables, two measurements were taken from two aliquots, and the average was used in statistical analyses. Repeatabability [55] was ≥0.95 (F_1, 31_>40.0, p<0.001) for concentration of MDA, soluble protein, and activity of GPX enzyme, and it was 0.71 (F_1, 31_ = 5.94, p<0.001) for SOD and 0.78 (F_1, 31_ = 13.96, p<0.001) for GR enzymes.

### Statistical Analyses

For statistical analyses, we performed Generalized Linear Mixed Effects Models of Restricted Maximum Likelihood (REML-GLMM) [56] by using the package “nlme” [57] in R [58]. In each model, nest of origin was introduced as a random factor to control for variance among nests, thus avoiding pseudoreplication [59]. We checked for the interaction between nest and treatment, which in all cases proved non-significant, and thus was removed from final models. The lack of a significant interaction implies that the effect of treatment was independent from that of nest. We included date of sampling as a covariate in every model, given that time of storage may affect enzymatic activity and protein concentration [60], and date may affect variables such as immune response [61] and MDA concentration [40]. Given that immune response, mass gained, MDA level, and treatment are supposed to be interrelated, when we search for the effect of treatment on MDA level, we controlled for immune response and mass gained, introduced as covariate. Similarly, in an additional model we looked for the relationship between MDA level and begging time, controlling for immune response and mass gained. For every model, we checked for homogeneity of variances (Levene’s test), and for normality of residuals by using the Kolmogorov-Smirnov test [56]. Means are given with their standard error (SE).

## Results

There were no significant differences in initial body mass, food ingested and feces mass excreted, between high begging (HB, *n* = 16) and low begging (LB, *n* = 16) nestlings (Table 1). Nestlings stimulated to beg every hour solicited food for a longer time in the HB treatment (67.8±4.17 s/hour on average) than in the LB treatment (5.0±0.58 s/hour; Table 1). HB nestlings mounted a smaller immune response to phytohaemagglutinin (smaller patagium swelling) than LB nestlings (Table 1). When we controlled for differences in immune response, mass gained was significantly lower in HB (6.0±1.1 g) than in LB nestlings (10.0±1.1 g; *F*_1, 17_ = 6.68, *P* = 0.019). Note that mass gained was negatively correlated with immune response (*β* = −0.35, *F*_1, 17_ = 4.71, *P* = 0.045).

**Table 1:** Mean ± SE for each variable measured in the study, and the effects of Treatment, controlling for Nest (random factor) and Date.

	HB (*n* = 16)	LB (*n* = 16)	Treatment
Initial body mass (g)	81.0±3.2	80.5±3.1	0.01^ns^
Consumed food (g)	98.8±3.7	97.5±3.6	0.00^ns^
Feces mass (g)	39.3±2.8	40.3±2.7	0.00^ns^
Mass gained (g)	6.6±1.3	9.0±1.2	2.79^ns^
Immune response (mm)	0.58±0.06	0.69±0.06	5.13*
Total time begging (s)	2930±115	446±122	211.42***
MDA (nmoles/ml)	31.3±2.6	29.6±2.5	0.42^ns^
SOD (mU/ml)	526±68	525±62	0.03^ns^
GPX (mU/ml)	596±63	690±60	3.15^ §^
GR (mU/ml)	62.6±8.6	63.0±8.3	0.00^ns^
Protein (mg/ml)	62.9±9.6	48.0±9.2	4.03^§^

A Restricted Maximum Likelihood Estimation General Linear Mixed Model (REML-GLMM) was used. *F*-values are shown. HB is for nestlings begging at a high level, and LB for nestlings begging at lower levels. Degrees of freedom were 1 for Treatment, and 18 for error. For time begging the analysis was performed after log-transformation, although raw data are shown. *P*-values: * for *P*<0.05; ** for *P*<0.01, and *** for *P*<0.001, § for 0.05<*P*<0.10, and ns for non significant.

**Figure 1:**
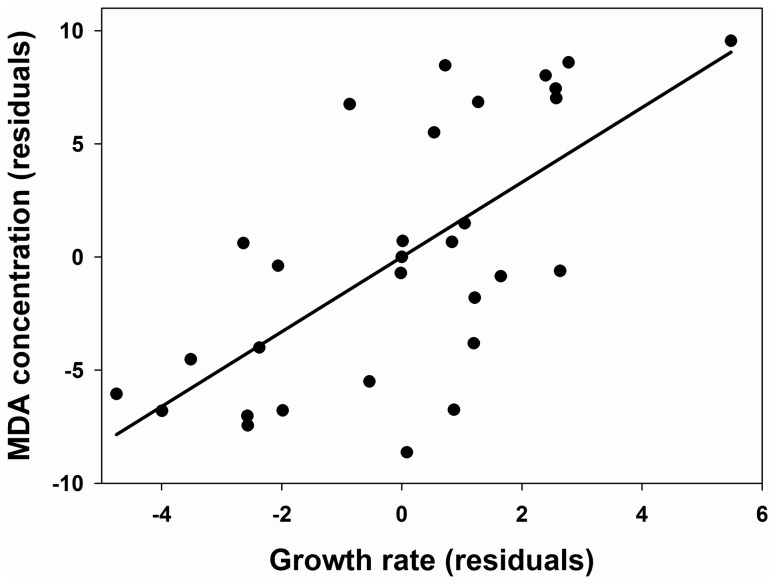
Relationship between MDA concentration (lipid peroxidation) and mass gained. The residuals, after controlling for treatment (time begging), nest (random), and date, are shown. The regression line is shown.

**Figure 2:**
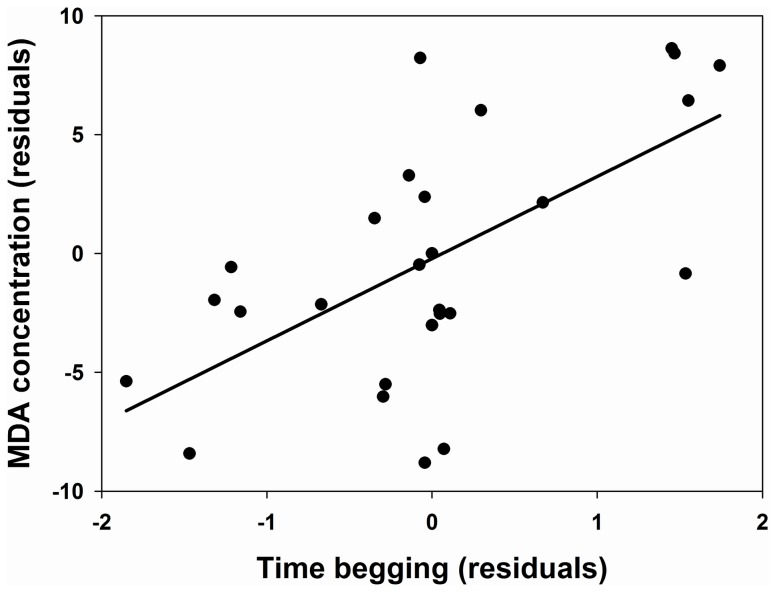
Relationship between MDA concentration (lipid peroxidation) and begging time. The residuals, after controlling for mass gained, immune response to phytohaemagglutinin, nest (random), and date, are shown. The regression line is shown.

On the other hand, there was no difference between both groups in the levels of MDA detected, nor in the concentrations of the antioxidant enzymes (Table 1). Nevertheless, when we controlled for mass gained and immune response, MDA concentration tended (*P* = 0.066) to be higher in HB (37.9±2.8 nmoles/ml) than in LB nestlings (29.6±2.1 nmoles/ml; Table 2). The model showed a positive correlation between mass gained and MDA (*β* = 0.37; Fig. 1). Moreover, when controlling for mass gained and immune response, the amount of time spent begging was positively correlated with MDA concentration (*β* = 0.25; Table 3; Fig. 2). When controlling for mass gained and immune response, protein concentration was significantly higher in HB (71.7±12.5 mg/ml) than in LB nestlings (43.6±9.4 mg/ml; Table S1). The experimental treatment had no effect on the activity of the three enzymes measured (Tables S2, S3, S4).

**Table 2:** Restricted Maximum Likelihood Estimation General Linear Mixed Model (REML-GLMM) showing the effect of treatment on concentration of malondialdehyde (MDA) as indicator of damage caused by oxidative stress, controlling for nest (random), date, mass gained and immune response to phytohaemagglutin.

	d.f.	*β*	*F*	*P*
Intercept	1		0.27	0.607
Treatment	1		3.88	0.066
Mass gained	1	0.37	6.61	0.021
Immune response	1	0.17	1.66	0.215
Date	1	0.85	4.88	0.042
Error	16			

**Table 3:** Restricted Maximum Likelihood Estimation General Linear Mixed Model (REML-GLMM) showing the relationship between time begging and concentration of malondialdehyde (MDA) as indicator of damage caused by oxidative stress, controlling for nest (random), date, mass gained and immune response to phytohaemagglutin.

	d.f.	*β*	*F*	*P*
Intercept	1		0.67	0.426
Time begging	1	0.25	5.45	0.036
Mass gained	1	0.35	5.97	0.030
Immune response	1	0.11	0.71	0.414
Date	1	0.86	5.04	0.043
Error	13			

## Discussion

At a first glance, there were not differences in levels of oxidative damage between HB and LB nestlings, but nestlings begging for longer reduced mass gained and immune response. When we controlled for mass gained and immune response, the positive correlation between begging time and oxidative stress emerged, which suggests that nestlings begging for longer reduced mass gain and immune response to avoid oxidative damage. Begging implies physical and neurological effort, which increases metabolism and thus RONS releasing [25], [26], [62]. Although several early studies failed to find higher metabolism in offspring begging more intensively, as measured by respirometry (O_2_ consumption) [63], [64], there is evidence that nestlings begging for longer show increased metabolism as a larger fraction of ingested energy spent in activity and maintenance and thus diverged from growth [21], [23], [31], [65]. If antioxidant defenses cannot combat RONS released by begging, oxidative stress might cause tissue damage in developing offspring, with negative long-term consequences [27]–[30]. In our study, the treatment had no effect on the activity of anti-oxidant enzymes. Nestlings might sustain high begging rates at low levels of oxidative stress by consuming non-enzymatic antioxidants, such as carotenoid or vitamin E [38], [39], but this only could be possible for healthy nestlings during a short period of time, before reserves of such non-enzymatic antioxidants are depleted.

On the other hand, nestlings might sustain high begging rates at low levels of oxidative stress by reducing other pro-oxidant components of life-history, such as growth and immune response. Both growing and mounting an immune response increase metabolic expenditure, contributing to RONS releasing. By reducing growth rate and immune response, nestlings may avoid immediate negative consequences of oxidative stress but, in turn, incurred other viability costs. Body size is an important determinant of survival in magpie nestlings and fledglings [44], [66], [67]. Therefore, nestlings reducing mass gained might face a higher mortality risk. Reduced immunocompetence also implies a survival cost for nestlings, as nestlings with lower immune responses have higher probabilities of dying [68]–[70].

HB nestlings could grow less than LB nestlings as a consequence of energy diverted to begging rather than as a way to reduce oxidative damage. However, this seems unlikely, because magpie nestlings showed flexible growth rates independently of begging intensity. HB nestlings grew less than LB nestlings at day 1 of the experiment, but they were growing at similar rates by day 3, despite begging intensity and food received remaining the same (Fig. S1). On day 3, HB nestlings returned to normal growth rates, but they did it at a cost in the way of increased oxidative stress (correlation between differences in mass gained between days 1 and 3, and MDA levels: *β* = 0.24; *F*_1, 13_ = 5.70, *P* = 0.03). This result suggests that reduction of mass gained at day 1 was a mechanism aimed at maintaining oxidative balance. In addition, there is compelling evidence showing that activities that are metabolically demanding, such as reproduction or physical exercise, may impair immune function in birds (e.g. [71]). A negative relationship between begging effort and immune response, probably mediated by steroid hormones, has been suggested for other species [33].

The existence of a viability cost associated with informative honest begging signals is necessary to explain the evolution of this behavior according to a number of models [2], [5], [72]. There is evidence of costs in the way of reduced growth [21], [23], [33] and immunocompetence in nestlings begging more intensively [31], [33]. However, nestlings begging more may receive extra food and thus compensate for the growth and immune costs, as suggested by a recent study [41]. In that study, nestlings were given a drug (cyproheptadine), which increases voluntary food intake in domestic fowl, pigeons and mammals [73], [74], with the aim of increasing begging intensity. Experimental chicks grew in a better body condition and showed enhanced immunocompetence at the end of the nestling period. The experimental treatment increased the probability of a nestling gaping and receiving food but, unfortunately, it failed to exert any effect upon time spent begging and postural intensity (the signal attributes likely to increase metabolic expenditure, hence physiological costs) [41], which casts doubt on its main conclusion that physiological costs of extra begging might be cancelled out by its benefits. While studies carried over the last decade ([21], [31]–[33], [41], this study) are beginning to unravel the types of physiological costs associated with begging signals, we are still far from a comprehensive field study where differences in begging effort (and their effects upon nestlings via parental response) can be mapped into direct fitness measures (e.g. recruitment rate or fecundity).

In conclusion, begging implies an immediate cost in terms of oxidative stress, but nestlings seem to circumvent this cost by incurring alternative ones, such as reduced growth and immunocompetence. By reducing growth and immune response, two processes generating pro-oxidants, nestlings maintained oxidative balance, which may have negative fitness consequences at older stages of the life cycle [75]. Therefore, physiological (growth and immunity) costs of begging seem to be mediated by oxidative stress, because fast-growing nestlings that have to beg intensively may not be able to sustain all these pro-oxidant functions simultaneously without suffering from oxidative damage. That is, nestlings show a three-way trade-off between begging, mass gained, and immune response in order to maintain oxidative balance.

## Supporting Information

Figure S1 **Variation in mass gained by magpie nestlings between day 1 and day 3 of the experiment.**
(PDF)

Table S1 **Model showing the effect of treatment on protein concentration.**
(PDF)

Table S2 **Model showing the effect of treatment on the concentration of active enzyme superoxide dismutase (SOD).**
(PDF)

Table S3 **Model showing the effect of treatment on the concentration of active enzyme glutathione peroxidase (GPX).**
(PDF)

Table S4 **Model showing the effect of treatment on the concentration of active enzyme gluthatione reductase (GR).**
(PDF)

Method S1 **Measurement of enzymatic activity.**
(PDF)
