# Supplementary material for: Oxidative Stress Mediates Physiological Costs of Begging in Magpie (Pica pica) Nestlings
Source: PLoS One. 2012 Jul 10;7(7):e40367. doi: 10.1371/journal.pone.0040367 (PMC3393730; doi:10.1371/journal.pone.0040367)
Supplement: Figure S1 — Variation in mass gained by magpie nestlings between day 1 and day 3 of the experiment. (PDF) [file pone.0040367.s001.pdf]

Oxidative stress mediates physiological costs of begging in magpie (*Pica pica*) nestlings

Gregorio Moreno-Rueda, Tomás Redondo, Cristina E. Trenzado, Ana Sanz, Jesús M. Zúñiga

Fig. S1. (A) Variation in mass gained by magpie nestlings between day 1 and day 3 of the experiment for the HB group (light grey) and the LB group (dark grey). Note that the day 1, the HB group showed smaller mass gained than HB group, but for the day 3, the two groups showed the same mass gained. (B) The difference in mass gained between the two days was positively correlated with the oxidative stress (MDA level), suggesting that nestlings increasing mass gained suffered a cost in the way of increased oxidative stress.

(A)

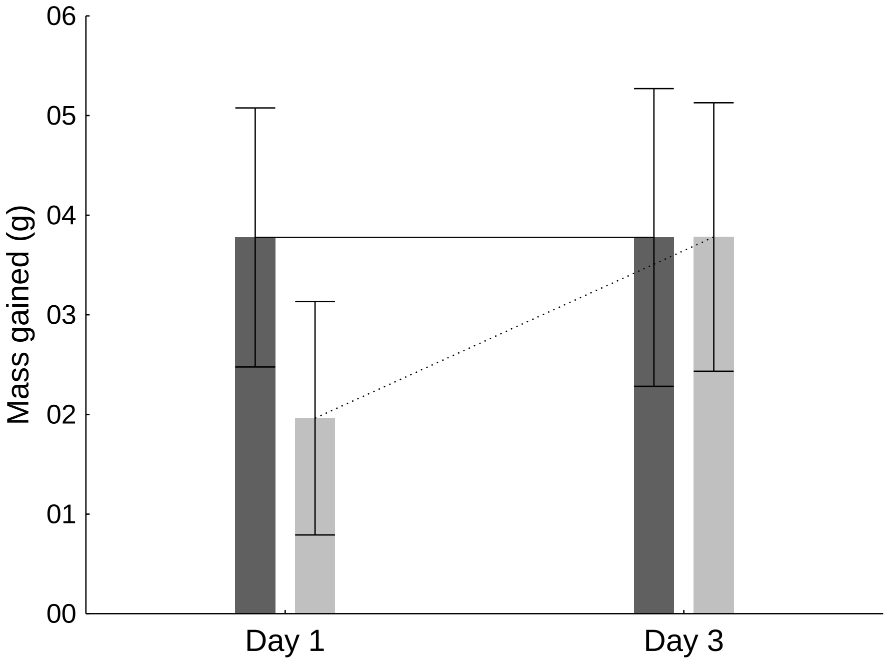

(B)

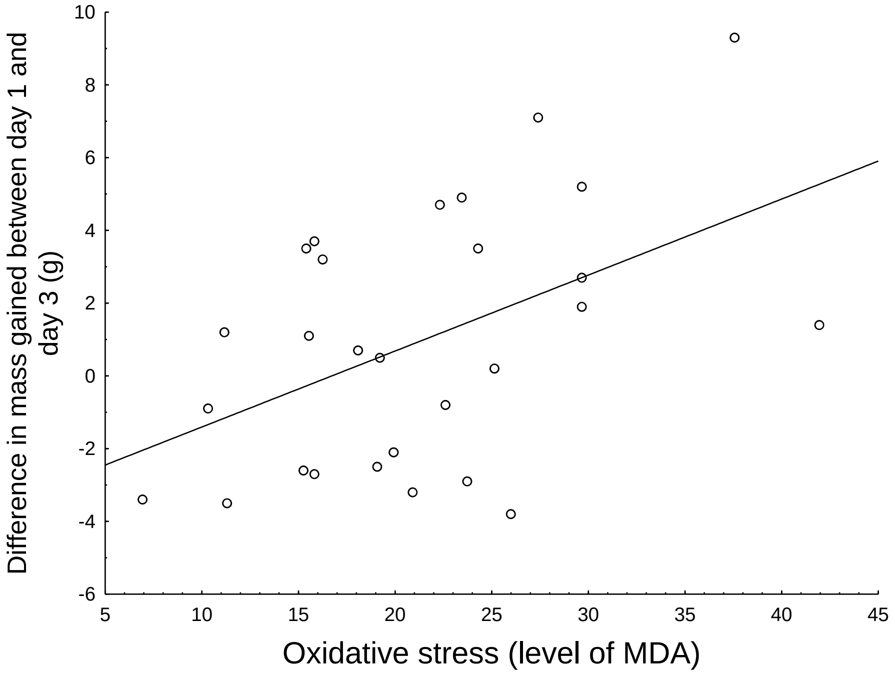
10
8
6
4
2
0
-2
-4
-6
5
10
15
20
25
30
35
40
45
Difference in mass gained between day 1 and day 3 (g)
Oxidative stress (level of MDA)
