# Supplementary material for: Oxidative Stress Mediates Physiological Costs of Begging in Magpie (Pica pica) Nestlings
Source: PLoS One. 2012 Jul 10;7(7):e40367. doi: 10.1371/journal.pone.0040367 (PMC3393730; doi:10.1371/journal.pone.0040367)
Supplement: Table S2 — Model showing the effect of treatment on the concentration of active enzyme superoxide dismutase (SOD). (PDF) [file pone.0040367.s003.pdf]

Oxidative stress mediates physiological costs of begging in magpie (*Pica pica*) nestlings

Gregorio Moreno-Rueda, Tomás Redondo, Cristina E. Trenzado, Ana Sanz, Jesús M. Zúñiga

Table S2. Restricted Maximum Likelihood Estimation General Linear Mixed Model (REML-GLMM) showing the effect of treatment on the concentration of active enzyme superoxide dismutase (SOD), after controlling for nest (random), date, growth rate and immune response to phytohaemagglutin.

	df	*t*	*P*
Intercept	16	3.83	0.002
Treatment	16	0.28	0.786
Date	16	3.83	0.002
Growth rate	16	1.05	0.308
Immune response	16	0.60	0.554
