# Supplementary material for: Oxidative Stress Mediates Physiological Costs of Begging in Magpie (Pica pica) Nestlings
Source: PLoS One. 2012 Jul 10;7(7):e40367. doi: 10.1371/journal.pone.0040367 (PMC3393730; doi:10.1371/journal.pone.0040367)
Supplement: Table S3 — Model showing the effect of treatment on the concentration of active enzyme glutathione peroxidase (GPX). (PDF) [file pone.0040367.s004.pdf]

Oxidative stress mediates physiological costs of begging in magpie (*Pica pica*) nestlings

Gregorio Moreno-Rueda, Tomás Redondo, Cristina E. Trenzado, Ana Sanz, Jesús M. Zúñiga

Table S3. Restricted Maximum Likelihood Estimation General Linear Mixed Model (REML-GLMM) showing the effect of treatment on the concentration of active enzyme glutathione peroxidase (GPX), after controlling for nest (random), date, growth rate and immune response to phytohaemagglutin.

	df	*t*	*P*
Intercept	16	1.04	0.316
Treatment	16	0.56	0.585
Date	16	1.04	0.316
Growth rate	16	1.65	0.119
Immune response	16	1.31	0.209
