# Supplementary material for: Oxidative Stress Mediates Physiological Costs of Begging in Magpie (Pica pica) Nestlings
Source: PLoS One. 2012 Jul 10;7(7):e40367. doi: 10.1371/journal.pone.0040367 (PMC3393730; doi:10.1371/journal.pone.0040367)
Supplement: Table S4 — Model showing the effect of treatment on the concentration of active enzyme gluthatione reductase (GR). (PDF) [file pone.0040367.s005.pdf]

Oxidative stress mediates physiological costs of begging in magpie (*Pica pica*) nestlings

Gregorio Moreno-Rueda, Tomás Redondo, Cristina E. Trenzado, Ana Sanz, Jesús M. Zúñiga

Table S4. Restricted Maximum Likelihood Estimation General Linear Mixed Model (REML-GLMM) showing the effect of treatment on the concentration of active enzyme gluthatione reductase (GR), after controlling for nest (random), date, growth rate and immune response to phytohaemagglutin.

	df	*t*	*P*
Intercept	16	2.63	0.018
Treatment	16	1.05	0.310
Date	16	2.63	0.018
Growth rate	16	0.25	0.805
Immune response	16	2.60	0.016
