# Supplementary material for: Oxidative Stress Mediates Physiological Costs of Begging in Magpie (Pica pica) Nestlings
Source: PLoS One. 2012 Jul 10;7(7):e40367. doi: 10.1371/journal.pone.0040367 (PMC3393730; doi:10.1371/journal.pone.0040367)
Supplement: Method S1 — Measurement of enzymatic activity. (PDF) [file pone.0040367.s006.pdf]

SUPPLEMENTARY INFORMATION

Oxidative stress mediates physiological costs of begging in magpie (*Pica pica*) nestlings

Gregorio Moreno-Rueda, Tomás Redondo, Cristina E. Trenzado, Ana Sanz, Jesús M. Zúñiga

Method S1: Measurement of enzymatic activity

All enzymatic assays were carried out at 25 ± 0.5 ºC using a PowerWavex microplate scanning spectrophotometer (Bio-Tek Instruments, USA) in duplicate in 96-well microplates (UVStar®, Greiner Bio-One, Germany).

Superoxide dismutase (SOD; EC 1.15.1.1) activity was measured spectrophotochemically by the ferricytochrome c method using xanthine/xanthine oxidase as the source of superoxide radicals. The reaction mixture consisted of 50 mM potassium phosphate buffer (pH 7.8), 0.1 mM EDTA, 0.1 mM xanthine, 0.013 mM cytochrome c and 0.024 IU ml^{-1} xanthine oxidase. One activity unit was defined as the amount of enzyme necessary to produce a 50% inhibition of the ferricytochrome c reduction rate measured at 550 nm [76].

Glutathione peroxidase (GPX; EC 1.11.1.9) activity was measured with a freshly prepared glutathione reductase solution (2.4 U ml^{-1} in 0.1 M potassium phosphate buffer, pH 7.0) was added to a 50 mM potassium phosphate buffer (pH 7.0), 0.5 mM EDTA, 1 mM sodium azide, 0.15 mM NADPH and 0.15 mM cumene hidroperoxide. After the addition of 1 mM GSH (reduced glutathione), the NADPH-consumption rate was monitored at 340 nm [77].

Glutathione reductase (GR; EC 1.6.4.2) activity was assayed by measuring the oxidation of NADPH at 340 nm [78]. The reaction mixture consisted of 0.1 M sodium phosphate buffer (pH 7.5), 1 mM EDTA, 0.63 mM NADPH, and 0.15 mM GSSG (oxidized glutathione).

For GPX and GR enzymatic activity, one unit of activity is defined as the amount of enzyme required to transform 1 μmol of substrate/min under the above assay conditions. Enzymatic activity of SOD is expressed in U/ml of hemolyzate, while for GPX and GR is expressed as mU/ml of hemolyzate. All biochemicals, including substrates, coenzymes, and purified enzymes, were obtained from Roche (Mannheim, Germany) or Sigma Chemical Co. (USA). All other chemicals came from Merck (Darmstadt, Germany) and were of the reagent grade.

References

76. McCord JM, Fridovich I (1969) Superoxide dismutase: an enzyme function for erythrocuprein. J Biol Chem 244: 6049-6055.
77. Flohé L, Günzler WA (1984) Assay of glutathione peroxidase. Methods Enzymol 105: 115-121.
78. Calberg I, Mannervik B (1975) Purification and characterization of the flavoenzyme glutathione reductase from rat liver. J Chem Ecol 250: 5475-5480.
